# Cannabis-Related Takotsubo Cardiomyopathy Presenting With Ventricular Tachycardia and Cardiogenic Shock Successfully Treated With Milrinone and Intra-Aortic Balloon Pump

**DOI:** 10.1016/j.jaccas.2025.104246

**Published:** 2025-05-19

**Authors:** Obaid I. Haque, Madiha Kiyani, Shahzad Hussain

**Affiliations:** a Medstar Health Georgetown University, Baltimore, Maryland, USA; b Pulmonary and Critical Care, MedStar Union Memorial Hospital, Baltimore, Maryland, USA

**Keywords:** cardiac assist devices, cardiomyopathy, left ventricle, systolic heart failure

## Abstract

A 63-year-old woman presented with intractable nausea, vomiting, dyspnea, and weakness after cannabis use. She had multiple prior admissions for cannabinoid hyperemesis syndrome. Upon arrival to the emergency department, she was hypotensive, tachycardic, and hypoxic. Laboratory results revealed an elevated troponin level (22,900 ng/L), NT-proBNP (21,092 pg/mL), lactic acidosis (lactic acid level of 7.1 mmol/L), and hypokalemia (potassium level of 2.6 mmol/L). The electrocardiogram showed ST-segment elevation in the anterior leads, and telemetry captured a wide complex tachycardia requiring cardioversion. The urine drug screening was positive for tetrahydrocannabinol. She was intubated and taken for urgent cardiac catheterization, which showed no coronary lesions. Right heart catheterization confirmed cardiogenic shock. She was initiated on norepinephrine, which was later transitioned to milrinone along with placement of an intra-aortic balloon pump for circulatory support. Transthoracic echocardiogram demonstrated a left ventricular ejection fraction of 15% to 20%, with mid to apical wall hypokinesis, which improved to baseline after 4 weeks of intensive management with milrinone and the intra-aortic balloon pump.

## Graphical Abstract

**Figure:**
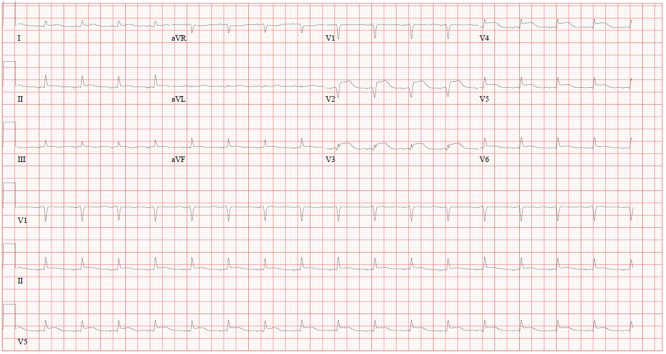

## Background

Takotsubo cardiomyopathy (TTC) or stress cardiomyopathy is a form of nonischemic cardiomyopathy that predominantly affects postmenopausal women and is characterized by acute and transient (<21 days) left ventricular systolic dysfunction in the absence of angiographically significant coronary artery disease often related to an emotional or physical stressful event.^1^^,^^2^ The left ventricular regional wall motion abnormalities characteristically extend beyond a single epicardial coronary artery distribution.^2^ The exact pathophysiology of Takotsubo syndrome remains to be elucidated; however, there is ample evidence to suggest that it involves catecholamine-mediated myocardial stunning, often precipitated by emotional or physical stressors.^1^, ^2^, ^3^, ^4^ Although cannabis is not yet traditionally recognized among the classic psychological or physical stressors, its use may provoke an adrenergic response similar to other physical or psychological triggers.^5^^,^^6^

**Take-Home Messages**

• This case adds to emerging evidence linking cannabis to TTC, particularly in patients with chronic conditions that heighten sympathetic sensitivity.
• Cardiac magnetic resonance is critical to exclude myocarditis and confirm TTC in complex cases.
• Early circulatory support, preferable with non-catecholaminergic agents, is essential for the management of cardiogenic shock in TTC.
• Intra-aortic balloon pump can be used to provide mechanical circulatory support in TTC with cardiogenic shock.

## History of Presentation

A 63-year-old Caucasian woman was brought into the emergency department by emergency medical services for a 2-day history of intractable nausea, vomiting, light-headedness, dyspnea at rest, and generalized weakness. On arrival to the emergency department, vitals were notable for a blood pressure of 94/60 mm Hg, a heart rate of 152 beats/min, a respiratory rate of 28 breaths/min, and oxygen saturation of 86% on room air. She endorsed regular marijuana use, last used 2 days before presentation, and denied any other recent physical or emotional stressor.

Physical examination was remarkable for cold extremities, jugular venous distention, bilateral lung crackles, and left ventricular gallop. Soon after the arrival, she had a transient loss of consciousness, during which telemetry showed wide complex tachycardia and she was cardioverted. She was initiated on supplemental oxygen for hypoxemia and respiratory distress; however, she continued to worsen and was intubated and placed on mechanical ventilation.

## Past Medical History

The past medical history was notable for multiple admissions for cannabinoid hyperemesis syndrome (CHS), paroxysmal atrial fibrillation, right lung adenosquamous carcinoma treated with robotic posterior segmentectomy and chemotherapy (carboplatin and Abraxane) 2 years ago, type 2 diabetes mellitus (managed with glimepiride and Ozempic), seropositive rheumatoid arthritis and Sjögren syndrome (managed with hydroxychloroquine 400 mg daily and sarilumab 200 mg biweekly), common variable immunodeficiency (managed with intravenous immunoglobulin every 21 days), and chronic obstructive pulmonary disease.

## Investigations

**Figure 1:**
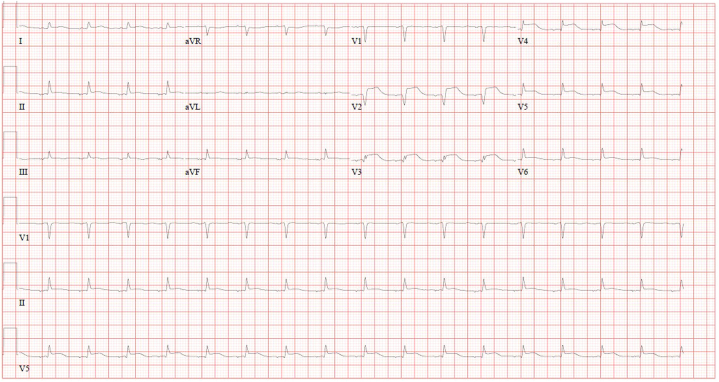
Electrocardiogram Showing ST-Segment Elevation in the Anterior Leads

Initial laboratory results were remarkable for severe hypokalemia, lactic acidosis, and a markedly elevated high-sensitivity cardiac troponin I and NT-proBNP levels. The electrocardiogram (ECG) was notable for ST-segment elevations predominantly in the anterior leads (Figure 1); hence, she was taken to the cardiac catheterization laboratory (Cath Lab) urgently for invasive angiography. The urine drug screen result was positive for tetrahydrocannabinol (THC). Laboratory results are summarized in Table 1.

**Table 1:** Initial Laboratory Results

Test	Result	Reference Range
Troponin I (high sensitivity) (ng/L)	22,900	0-30
NT-proBNP (pg/mL)	21091.0	900
Serum creatinine (mg/dL)	2.08	0.72 (baseline)
Total bilirubin (mg/dL)	0.9	0.3-1.2
ALT (units/L)	36	10-49
AST (units/L)	131	0-33
Potassium (mmol/L)	2.6	3.5-5.1
Lactic acid (mmol/L)	7.1	0.5-2.0
White blood cells (k/μL)	16.7	4.0-10.8
Differentials white cell count (%)		
Neutrophils	68.0	43-75
Lymphocyte	18.2	15-45
Monocyte	13.1	3-12
TSH (IU/mL)	0.257	0.55-4.7
Free T4 (ng/dL)	0.48	0.89-1.76

ALT = alanine aminotransferase; AST = aspartate aminotransferase; TSH = thyroid-stimulating hormone.

**Figure 2:**
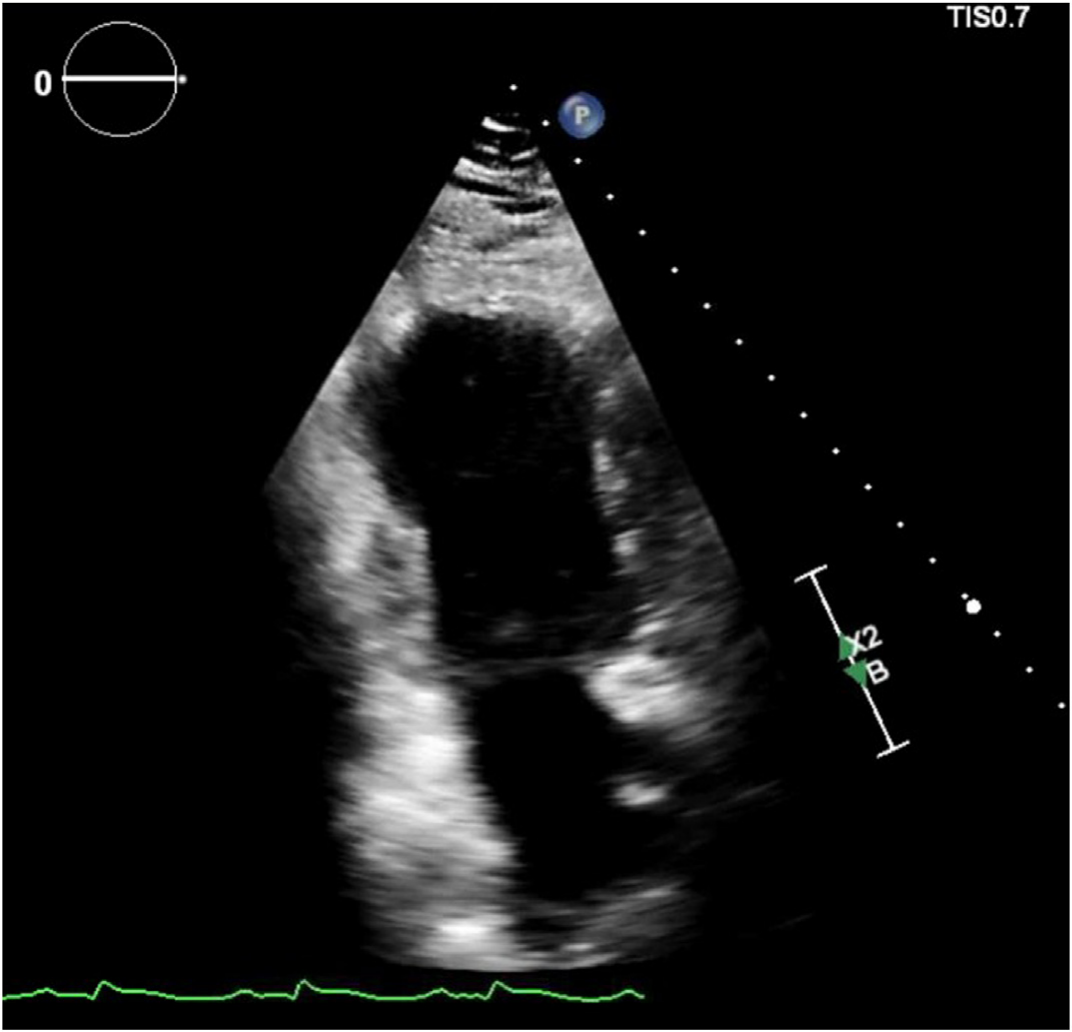
Echocardiogram Showing Apical Ballooning of the Left Ventricle

The coronary angiogram did not show any obstructive coronary lesions, and right heart catheterization revealed an elevated pulmonary capillary wedge pressure of 30 mm Hg, a cardiac index of 1.4 L/min/m^2^, and an elevated right atrial pressure of 15 mm Hg, consistent with new heart failure and cardiogenic shock of nonischemic etiology. The echocardiogram showed a left ventricular ejection fraction (LVEF) of 15% to 20%, with mid to apical wall hypokinesis (Figure 2, Video 1).

**Figure 3:**
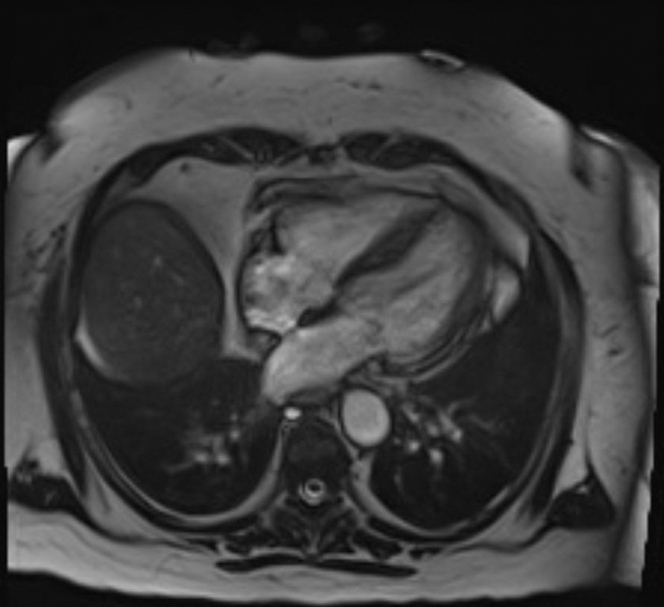
Cardiac Magnetic Resonance With Gadobutrol Contrast Showing Normal Left Ventricular Size With Preserved Left Ventricular Ejection Fraction and Hypokinesis of the Apical Segment of the Left Ventricle

Cardiac magnetic resonance performed on day 13 of presentation showed normal left ventricular size with preserved overall ejection fraction and apical hypokinesis (Figure 3). There were large areas of increased T1 and T2 signal consistent with acute myocardial edema, without significant late gadolinium enhancement. A small area of basal lateral contrast uptake suggested minimal fibrosis or a prior infarct.

## Differential Diagnosis

Given the markedly elevated cardiac troponin levels, ST-segment elevation on ECG, and cardiogenic shock, she was initially diagnosed with ST-segment elevation myocardial infarction; however, the left heart catheterization ruled against it. The leukocytosis on presentation and past medical history of extensive autoimmune diseases raised the suspicion of myocarditis. The cardiac magnetic resonance performed on day 13 did not show any findings consistent with myocarditis.

## Treatment

She was intubated and mechanically ventilated and taken into the Cath Lab, her blood pressure continued to drop, and she was initiated on inopressor support with norepinephrine and mechanical circulatory support with an intra-aortic balloon pump (IABP). On day 2, vasopressor support was transitioned to milrinone for suspected TTC. By day 5, with improved hemodynamics, the IABP was removed and she was extubated, though milrinone was continued until day 10. She was given a trial of valsartan/sacubitril for heart failure management, which resulted in symptomatic hypotension, necessitating its discontinuation.

## Outcome and Follow-Up

The patient's hemodynamic status improved gradually with intensive supportive care. Serial imaging demonstrated recovery of ventricular function, and the final diagnosis of TTC was confirmed based on cardiac magnetic resonance. On a follow-up echocardiogram performed 4 weeks later, she had complete recovery of LVEF; however, her baseline global longitudinal strain was mildly impaired at −11%.

## Discussion

This report describes rare case of TTC in a postmenopausal woman with a chronic cannabis use who developed cardiogenic shock with CHS. TTC is characterized by transient left ventricular dysfunction in the absence of obstructive coronary disease, typically triggered by physical or emotional stress. Emerging evidence implicates cannabis use as a potential precipitant. Our patient with a history of CHS presented with gastrointestinal symptoms after cannabis use, was found to have severe hypokalemia secondary to intractable vomiting, elevated troponin and NT-proBNP levels, ST-segment elevations on ECG, markedly reduced ejection fraction with left ventricular wall motion abnormalities, and a negative coronary angiography result. Cardiac magnetic resonance performed 2 weeks later demonstrated normalization of the ejection fraction, persistent apical hypokinesis with acute edema, and no contrast uptake consistent with TTC. Notably, she had no identifiable physical or emotional stressor preceding the presentation.

The physiological effects of cannabis are primarily mediated through the interaction of THC with the endocannabinoid systems. The endocannabinoid system consists of the endogenous ligands N-arachidonoyl-ethanolamine and 2-arachidonoylglycerol, their target cannabinoid receptor types 1 and 2 (CB1 and CB2), and their synthetic and catabolic enzymes.^5^ The endocannabinoid system plays a significant role in modulating emotional behaviors and stress response through the hypothalamic-pituitary-adrenocortical axis.^5^ The CB1 receptor is extensively expressed within the central (including several limbic regions—hippocampus, amygdala, and prefrontal cortex), peripheral sensory, and autonomic nervous systems and is the primary target of THC.^7^ A significant proportion of the cardiovascular effects of cannabinoids are thus mediated through activation of the sympathetic nervous system and inhibition of the parasympathetic autonomic nervous system leading to a hyperadrenergic state.^5^^,^^6^^,^^8^ In addition, cannabinoids have been shown to decrease myocardial contractility in humans via CB1 receptors in a dose-dependent manner.^7^ Cannabis, an emerging and less conventional trigger, likely precipitated a catecholamine surge in this patient. The patient's pre-existing comorbidities (rheumatoid arthritis, Sjögren syndrome, type 2 diabetes, and recent atrial fibrillation ablation) likely compounded the myocardial vulnerability.

The patient presented with cardiogenic shock, necessitating inopressor support. Initially, norepinephrine was used; however, given the suspicion for TTC and its pathophysiology linked to catecholamine excess,^4^ it was transitioned to milrinone as it acts through a noncatecholaminergic mechanism. In addition, IABP was used to provide mechanical circulatory support. There is a lack of prospective randomized studies, and no clear guidelines exist for the management of cardiogenic shock in TTC. Catecholamine excess is central to TTC pathophysiology, and catecholaminergic inotropes have been shown to increase short- and long-term mortality.^9^^,^^10^ Non-catecholaminergic inotropic agents such as levosimendan and milrinone are preferred in cardiogenic shock secondary to TTC.^10^^,^^11^ To our knowledge, this is the first reported case of TTC-related cardiogenic shock successfully managed with IABP and milrinone.

Regarding long-term management, angiotensin-converting enzyme inhibitors or angiotensin receptor blockers have been associated with improved 1-year survival and reduced recurrence risk.^10^ Beta-blockers have not shown any mortality benefit in TTC; evidence is lacking for statins, sodium-glucose cotransporter 2 (SGLT2) inhibitors, and glucagon-like peptide-1 (GLP-1) receptor agonists, while aspirin has been linked to increased adverse outcomes and is not routinely recommended.^10^ Our patient was started on sacubitril/valsartan but could not tolerate it. Avoiding triggers (cannabis in this case) and treatment of underlying psychiatric disorders might help in preventing recurrences.

Although historically considered benign, TTC is increasingly recognized as a condition with significant morbidity. Our patient presented with ventricular tachycardia and cardiogenic shock. Recent observational data suggest that serious in-hospital complications such as cardiogenic shock and death rates in TTC are comparable to patients with acute coronary syndrome.^12^ Predictors of adverse in-hospital outcomes include the presence of physical triggers, acute neurologic or psychiatric diseases, initial troponin >10 times the upper normal limit, and LVEF <45%.^12^

## Consent

Written consent was obtained before publication.

## Funding Support and Author Disclosures

The authors have reported that they have no relationships relevant to the contents of this paper to disclose.

## Appendix

Video 1 Trans-thoracic electrocardiogram showing severe mid-to-apical hypokinesis and apical ballooning of the left ventricle.
